# Stress Monitoring on GFRP Anchors Based on Fiber Bragg Grating Sensors

**DOI:** 10.3390/s19071507

**Published:** 2019-03-28

**Authors:** Hai-Lei Kou, Wang Li, Wang-Chun Zhang, Yuan Zhou, Xiao-Long Zhou

**Affiliations:** 1 College of Engineering, Ocean University of China, Qingdao 266100, China; liwang@stu.ouc.edu.cn; 2 Cooperative Innovation Center of Engineering Construction and Safety in Shandong Blue Economic Zone, Qingdao University of Technology, Qingdao 266033, China; 3 School of Architecture and Civil Engineering, Weifang University of Science and Technology, Weifang 262700, China; zhangwangchun@wfust.edu.cn; 4 Facility Horticulture Laboratory of Universities in Shandong, Weifang University of Science and Technology, Weifang 262700, China; 5 College of Environmental Science and Engineering, Ocean University of China, Qingdao 266100, China; zy2057@stu.ouc.edu.cn; 6 Construction Engineering College, Qingdao Agriculture University, Qingdao 266109, China; b20130013@xs.ustb.edu.cn

**Keywords:** glass fiber-reinforced polymer (GFRP) anchor, fiber bragg grating (FBG) technology, field test, axial force, shear stress

## Abstract

Glass fiber-reinforced polymer (GFRP) bolts have been widely used in some applications of grouted anchors because of the advantages of better resistance to corrosion, high strength-to-weight ratio, low electromagnetic properties, and so on. This study presents a field test to assess the feasibility of fiber Bragg grating (FBG) sensors in monitoring the stress profile of GFRP anchors during pulling test. Two GFRP anchors were fully instrumented with FBG sensors and then installed into the ground using a drilling and grouting method. To measure the stress profile along test anchors, seven bare FBG sensors were arranged in a single optical fiber and then embedded in the middle of GFRP bolts in the process of extrusion molding. The procedure for embedding bare FBG sensors into GFRP bolts is introduced first. Then, the axial forces and shear stresses that were calculated from the measurements of the FBG sensors are discussed. The field test results indicate that the embedded FBG technology was feasible to monitor the stress state of GFRP anchors during pulling.

## 1. Introduction

Glass fiber-reinforced polymer (GFRP) materials have been increasingly used in civil engineering in the past few decades [1], such as GFRP rebars in concrete beams, strengthening of concrete girders with epoxy-bonded GFRP plates, GFRP connections, fire protection of GFRP pultruded profiles, and so on [2,3,4,5]. GFRP materials have the advantages of better resistance to corrosion, high strength-to-weight ratio, and low electromagnetic properties, and GFRP bolts have been an alternative to steel in some applications of grouted anchor bolts [6]. In this system, antipulling tests are vital for understanding the actual field behavior of anchors for determining the relevant geotechnical parameters [7,8]. Although a wealth of load–displacement curves of GFRP anchors are nowadays available, the ability to acquire information along the anchor depth opened more opportunities for studying the real interaction between GFRP anchor and soils [6,8].

Appropriate instrumentation is crucial for GFRP anchors so that useful and reliable parameter values are obtained for the designers [9,10]. Traditional sensors, such as electrical resistance strain gauges, dial gauges, and extensometers are not feasible for GFRP pultruded profiles as the materials are anisotropic and are not easily instrumented [6,11]. Recent development in optical fiber technology have provided an excellent choice to civil engineers because of the small dimensions, good resolution and accuracy, wide temperature operating range, and excellent ability to transmit signal over long distances [12]. For geotechnical application, Li et al. [13] used fiber Bragg grating (FBG) sensors to monitor the ground movement for land slide and reveal the potential safety issue [14,15]. Hong et al. [16] adopted multiplexed FBG sensors to assess the performance of soil nails during pulling. Schilder et al. [17] measured the strain distribution of bored piles using FBG sensors and examined the load transfer mechanism. Similar studies were also conducted by Lee et al. [18], Kong et al. [19], and Wang et al. [20]. The researches mentioned above usually directly adhered FBG sensors on the pregrooved surface of steel bar and covered it with epoxy resin [21,22,23]. For GFRP bar, Zhang et al. [15], Pei et al. [24], Li et al. [25], and Jin et al. [26] also adopted the method of adhering bare FBG sensors straightly along a precreated groove to monitor the axial strain. Then, epoxy resin or glue was used to cover the whole sensor section. However, their method could ruin GFRP anchors and affect the results accuracy. Hence, there is a practical demand to search a feasible method for strain gauges installation and investigate the real interaction behavior of GFRP anchor and soil.

In this paper, a novel method based on FBG sensors is proposed and its application on monitoring the behavior of GFRP anchors is also presented. One line of optical fiber multiplexed with bare FBG sensors was embedded into GFRP anchors in the process of extrusion molding. On this basis, the axial forces at different levels were determined during pulling. Thus, the shear stress profiles along GFRP anchors could be calculated.

## 2. Field Test Procedures

### 2.1. Implantation of Bare FBG Sensors into GFRP Anchors

**Figure 1:**
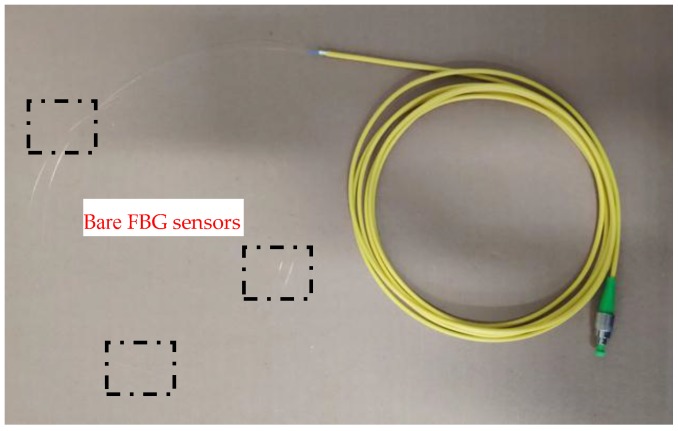
Fiber line with bare FBG sensors.

The bare FBG sensors, manufactured by Ningbo Shangong Center of Structural Monitoring, China, were fused with an optical fiber at planned intervals to form a line of bare FBG sensors, as shown in Figure 1. Each bare FBG sensor had a different wavelength in the range of 1510 to 1590 nm. The bigger wavelength differences were used for adjacent sensors to avoid wavelength overlap. Then, the optical fiber line was placed in the middle of anchor mold and fixed on its end tightly. When injecting the resin into the mold, the optical fiber line could be fused together with GFRP material to form a smart GFRP anchor. After that, the mold was cured at 60 °C for 24 h and then for another 24 h at 85 °C to form a GFRP anchor. This installation method can avoid material damage, and therefore ensures the integrity of GFRP anchors. More detail information of the used FBG sensors is shown in Table 1.

**Figure 2:**
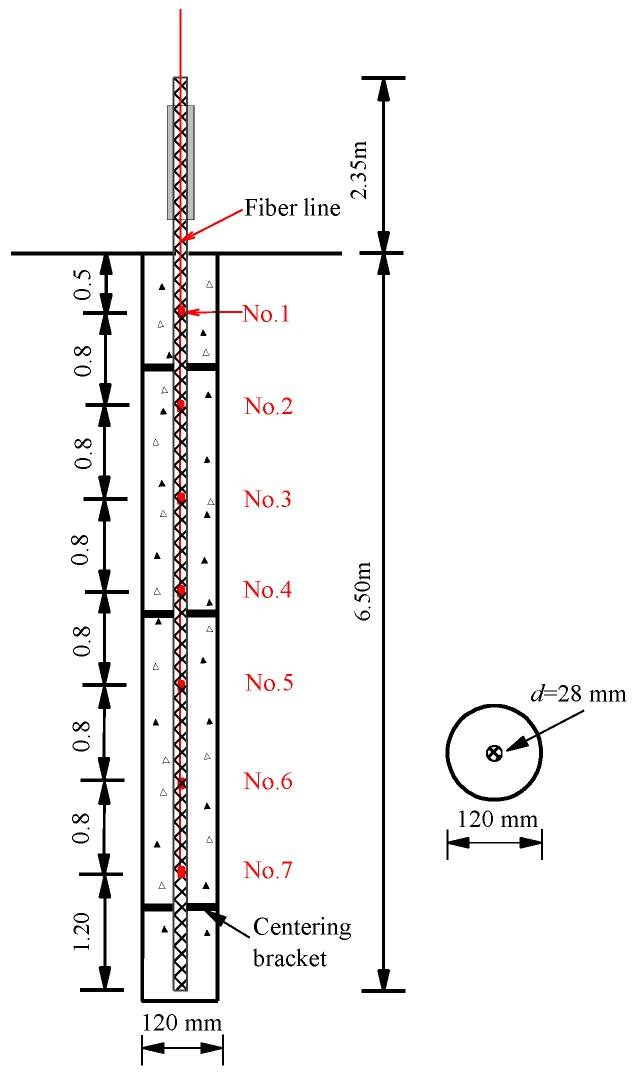
Schematic diagram of instrumented glass fiber-reinforced polymer (GFRP) anchor.

Two GFRP anchors—GFRP-1 and GFRP-2—both with a length of 8.85 m and a 28 mm diameter were instrumented with FBG sensors. Seven bare FBG sensors were embedded in each GFRP anchors, as shown in Figure 2. In order to measure the stress change near anchor head, the distance from the uppermost sensor to the ground surface was 0.5 m. Other strain sensors were arranged at 0.8 m intervals along the tested anchors, as illustrated in Table 1. The density of the used GFRP anchors was 2.1 g/mm^3^. The Young’s modulus and ultimate tensile strength were 45.0 GPa and 750 MPa, respectively.

### 2.2. Installation of GFRP Anchors

The field test was conducted at a construction site near Ocean University, China. A subway station project was to be built at this site. The subsoil was 0.0–15.0 m deep moderately decomposed granite (MDG). The unit weight of the MDG layer is 24.5 kN/m^3^, the effective friction angle is 55°, and the elasticity modulus is 32.0 MPa. After the GFRP anchors were embedded with all FBG sensors, a preliminary check was conducted in factory to inspect the survival rate of FBG sensors. Calibration was also carried out in the factory to determine the strain sensitivity of installed FBG sensors before field application. Thereafter, the GFRP anchors were transported to the field for installation.

**Figure 3:**
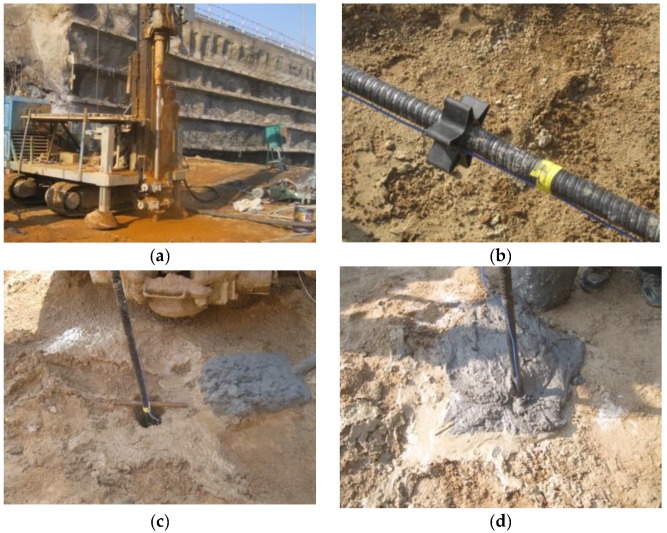
Installation procedure for GFRP anchors: (**a**) drilling hole; (**b**) centering; (**c**) installing; and (**d**) pouring cement mortar.

The GFRP anchors were installed with drilling and grouting method. The schematic representation and installation in field are shown in Figure 2 and Figure 3, respectively. The drilling direction was perpendicular to the ground and was drilled by a pneumatic rotary percussion drilling machine. According to the actual situation of the test site, the borehole diameter was 120 mm and the depth was 6.5 m (Figure 3a). In order to avoid the influence of insufficient anchor spacing on the test results, it was necessary to reserve more than 1.0 m between two test anchors when installed. After the holes were finished, the test anchors were tied to several centering brackets to ensure that the GFRP anchor was placed in the middle of the hole, as shown in Figure 3b. Then, the GFRP anchors were placed into the drill holes centrally (Figure 3c). After that, Grade M32.5 cement mortar was poured around each anchor to enhance the GFRP anchors with the surrounding soils (Figure 3d).

### 2.3. Pullout Testing Setup

**Figure 4:**
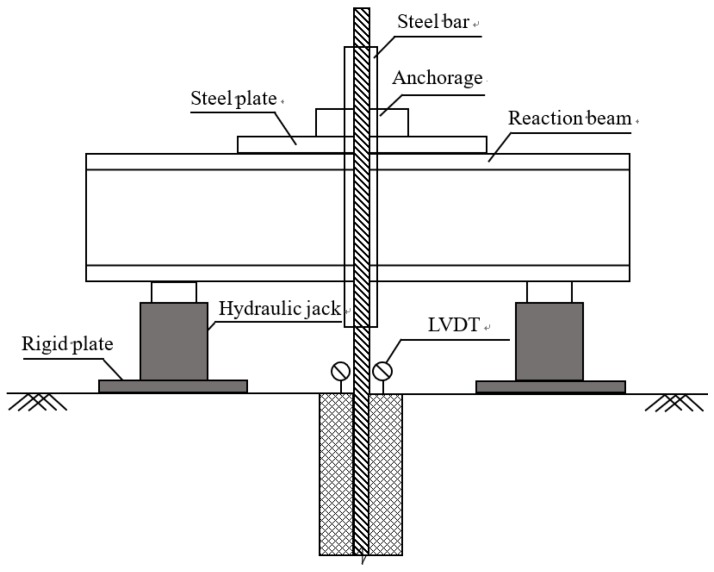
Schematic diagram of pulling test.

**Figure 5:**
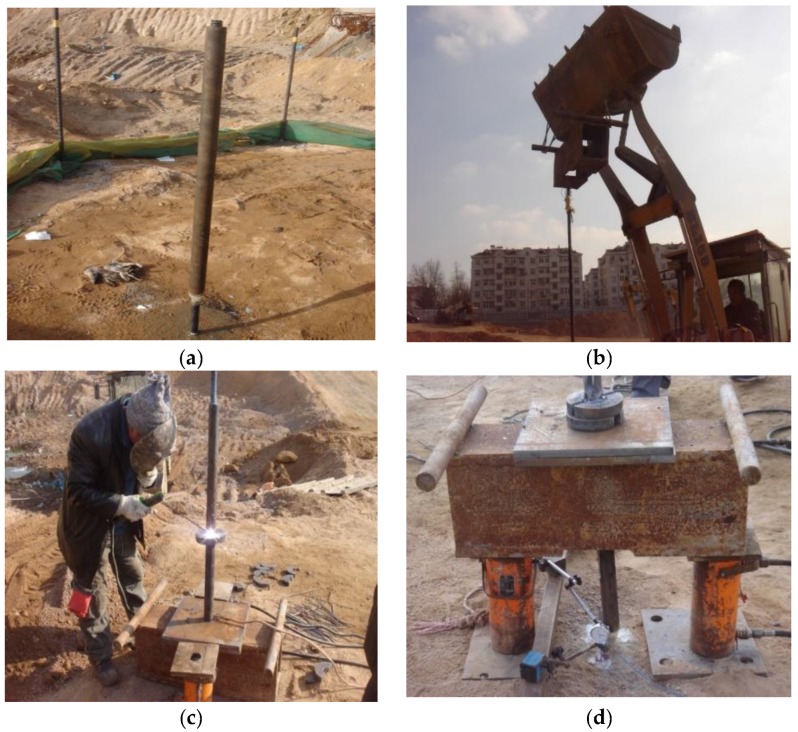
Pulling test set-up in field: (**a**) steel bar fixed with GFRP anchors; (**b**) loading device installation; (**c**) wedding circular steel plates; and (**d**) pulling-out.

The pulling out tests were conducted 28 days after GFRP anchor installation. Figure 4 and Figure 5 show the test setup schematic diagram and instrumentation in field. In order to avoid the crushing failure of GFRP anchor during pulling, a steel bar with 32.0 mm inner diameter, 10.0 mm wall thickness, and 1.0 m length was used to reinforce the exposed portion of the anchors and connect the anchors to the reaction beam, as shown in Figure 5a. The used loading device was specially processed in a factory and lifted by one excavator in-field to pass through the GFRP anchors (Figure 5b). Two circular steel plates with 4.0 cm thickness were tightly fixed with the steel bar by welding method in order to apply the reaction force (Figure 5c). At the bottom of each hydraulic jack, one rigid cushion plate with 3.0 cm thickness was placed to prevent the uneven settlement of the soil under jacks during loading. One pressure gauge and two linear variable differential transformers (LVDT) were adopted to measure the pulling force and displacement in the test (Figure 5d). The pullout tests were conducted according to ASTM D4435-13e1 [27]. A SI425 interrogator system connected with a laptop computer was used to record the values of embedded FBG sensors in real-time.

## 3. Test Results and Discussion

### 3.1. Axial Forces Along Depth

The wavelength shifts of each FBG sensor can be obtained using the recorded wavelength *λ_B_*. Using Equation (1), the axial strain *ε_i_* of each FBG sensor under different pulling forces can be calculated [28]:

*ε_i_* = {(*Δλ_B_*/*λ_B_*_0_) − *Κ_t_ Δt*}/*K_ε_*, (1)

where *Δλ_B_* is the wavelength shift of FBG sensors; *λ_B_*_0_ is the initial wavelength of each FBG sensor before loading; *K_ε_* is the strain sensitivity coefficient of FBG sensors, which equals 1.2 pm/*με* for the sensors used in this study; *Κ_t_* is the temperature sensitivity coefficient of FBG sensors; and *Δt* is the temperature variation during pulling process. Though the field tests were performed on a cold day, the temperature variances were not considered as the pulling process was completed in a few minutes. It should be noted that the values of all FBG sensors were initialized before loading, and thus the measured strain only reflects the interaction between the GFRP anchor and the soil.

Once the axial strain *ε_i_* is known, the axial force *F_i_* can be calculated using the method proposed by [29].

*F_i_* = *E A ε_i_*, (2)

where *E* is the Young’s modulus of the test GFRP anchor, which equals 45.0 GPa in this study, and *A* is the cross-sectional area defined by diameter *D* = 28.0 mm.

**Figure 6:**
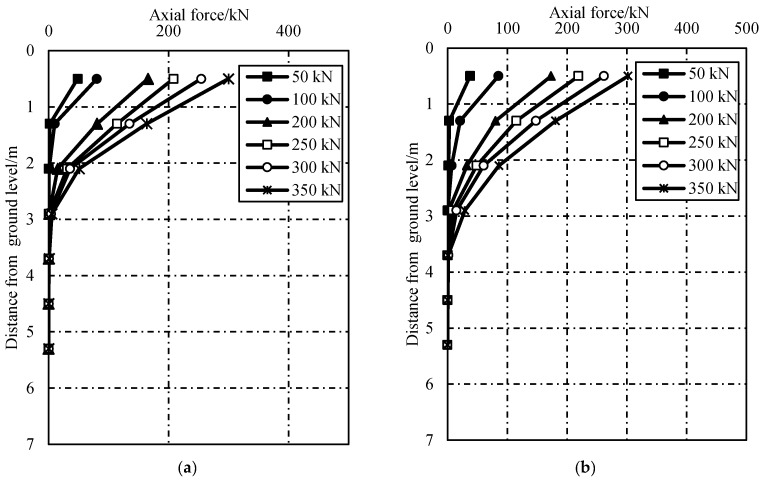
Axial forces versus depth: (**a**) GFRP-1 and (**b**) GFRP-2.

The axial forces along test anchors can be calculated using Equation (2), as shown in Figure 6. It indicates that the axial forces of GFRP-1 and GFRP-2 decrease with depth under a certain pulling force. For the pulling force of 50 kN, the axial forces distributions were approximately linear. This shown that the shear stress along the anchors was also uniformly distributed. However, with the increase of pulling forces, the shear stresses of GFRP–cement interface were gradually mobilized and the distribution became nonlinear. The maximum axial forces of GFRP-1 and GFRP-2 measured in pulling were about 300 kN. As the Young’s modulus of the GFRP bars were 45.0 GPa, the ultimate tensile strength should be 3078 kN. Therefore, as the measured values were much smaller than the calculated limits, it is likely that only local failures took place.

### 3.2. Average Shear Stress Along Depth

The average shear stress can be obtained from the relationship of axial forces [30]:

*f* = (*F_i_* − *F_i-_*_1_) / [*π D*(*h_i_* − *h_i-_*_1_)] = [*E D*(*ε_i_* − *ε_i-_*_1_)]/4 (*h_i_* − *h_i-_*_1_), (3)

where *F_i_*, *F_i-_*_1_ are the axial forces at levels *i* and *i*-1, respectively, which can be calculated using Equation (2); (*h_i_ − h_i−_*_1_) is the distance of FBG sensors between levels *i* and *i*-1; and *D* is the diameter of the GFRP anchor used.

**Figure 7:**
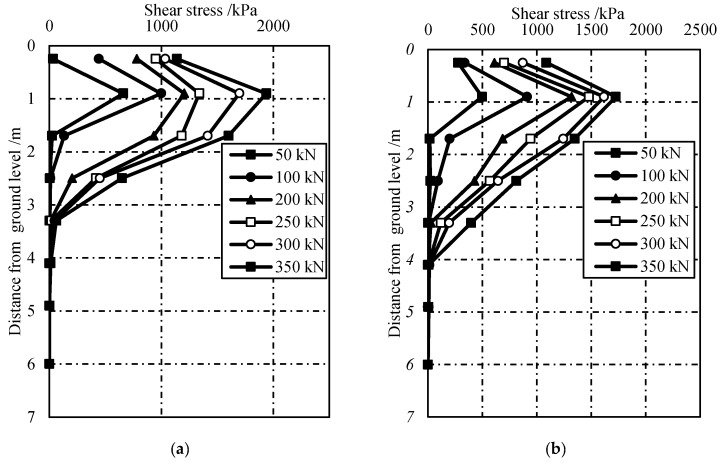
Shear stress versus depth: (**a**) GFRP-1 and (**b**) GFRP-2.

The calculated shear stresses using Equation (3) under different pulling forces are shown in Figure 7. It is obvious that the shear stress at lower pulling force is smaller than that of larger pulling force. The peak shear stress was ~1.94 and ~1.72 MPa for GFRP-1 and GFRP-2, respectively. These values were much smaller than that of the ultimate tensile strength of GFRP anchors, which was ~750 MPa. From this perspective, it can be deduced that the interlaminar shear is the main failure model for GFRP anchor system. This is different from the failure model in steel anchor system, which usually fails at the grout–soil interface [11]. For GFRP anchors system, the interlaminar shear strength of GFRP materials is a very important parameter to evaluate the pullout resistance in GFRP anchor system.

It also can be seen from Figure 7 that there exists a critical load transferring depth for GFRP anchor system. Little shear stress could be transferred to the GFRP–cement interface below 4.0 m. That is, the critical load transferring depth for test anchors in this study was approximately 4.0 m. Hence, the ultimate pullout force of GFRP anchor system depends on the critical depth if the anchorage length was longer than this critical depth. This can be explained by the lower GFRP stiffness, which made the anchors more extensible and caused large strains. The value of this critical depth is probably closely related to the interlaminar shear strength of used materials, the geometry of bolts, and the surrounding soil characteristics.

## 4. Conclusions

In this paper, a novel method for monitoring the stress profile of GFRP anchor using embedded FBG sensing technology was introduced. Two GFRP bars were instrumented with bare FBG sensors and installed with drilling and grouting methods in-field. The following conclusions can be drawn.

1. The embedded FBG sensing technology was proven to be feasible in measuring the stress distribution of GFRP anchors during pulling. Optical fiber with multiplexed bare FBG sensors could be fused together well with GFRP materials in the process of extrusion molding to from smart monitoring system.
2. For the GFRP anchor system, the anchor would reach failure when the shear stresses near GFRP–grout interface exceeded the interlaminar shear strength, as they are anisotropic and more extensible. A critical depth exists for the GFRP anchor system. Therefore, the ultimate capacity of these structures will be unaffected by their depth if the length is greater than this critical value.

## Figures and Tables

**Table 1:** Details of the fiber Bragg grating (FBG) sensors used.

FBG Number	No. 1	No. 2	No. 3	No. 4	No. 5	No. 6	No. 7
**Initial Wavelength (nm)**	1517.17	1519.87	1525.42	1530.01	1534.98	1539.84	1544.55
**Distance from Ground Level (m)**	0.5	1.3	2.1	2.9	3.7	4.5	5.3

## References

[1] Chaallal O., Benmokrane B. (1993). Physical and mechanical performance of an innovative glass-fiber-reinforced plastic rod for concrete and grouted anchorages. Can. J. Civ. Eng..

[2] Saadatmanesh H., Ehsani M.R. (1989). Application of fiber-composites in Civil Engineering. Proceedings of the Sessions Related to Structural Materials at Structures Congress’ 89.

[3] Correia J.R. (2008). GFRP Pultruded Profiles in Civil Engineering: Hybrid Solutions, Bonded Connections and Fire Behavior. Ph.D. Thesis.

[4] Gonilha J.A., Correia J.R., Branco F.A. (2013). Creep response of GFRP-concrete hybrid structures: Application to a footbridge prototype. Compos. Part B Eng..

[5] Nye T.K., Pantelides C.P., Burningham C.A. (2018). Unidirectional GFRP composite connections between precast concrete wall panels under simulated seismic loads. Compos. Struct..

[6] Carvelli V., Fava G., Pisani M.A. (2009). Anchor system for tension testing of large diameter GFRP bars. J. Compos. Constr..

[7] Benmokrane B., Masmoudi R. (1996). Flexural response of concrete beams reinforced with FRP reinforcing bars. Struct. J..

[8] Carozzi F.G., Poggi C., Bertolesi E., Milani G. (2018). Ancient masonry arches and vaults strengthened with TRM, SRG and FRP composites: Experimental evaluation. Compos. Struct..

[9] Ashford S.A., Jakrapiyanun W. (2001). Drivability of glass FRP composite piling. J. Compos. Constr..

[10] Ozkal F.M., Polat M., Yagan M., Ozturk M.O. (2018). Mechanical properties and bond strength degradation of GFRP and steel rebars at elevated temperatures. Constr. Build. Mater..

[11] Kou H.L., Guo W., Zhang M.Y. (2015). Pullout performance of GFRP anti-floating anchor in weathered soil. Tunn. Undergr. Space Technol..

[12] Tennyson R.C., Coroy T., Duck G., Manuelpillai G., Mulvihill P., Cooper D.J., Jalali S.J. (2000). Fibre optic sensors in civil engineering structures. Can. J. Civ. Eng..

[13] Li J., Correia R., Chehura E., Staines S., James S.W., Tatam R.P. A fibre bragg grating-based inclinometer system for ground movement measurement. Proceedings of the Fourth European Workshop on Optical Fibre Sensors, International Society for Optics and Photonics.

[14] Yin J.H., Zhu H.H., Jin W. (2008). Monitoring of soil nailed slopes and dams using innovative technologies. Proceedings of the 10th International Symposium on Landslides and Engineered Slopes.

[15] Zhang C.C., Zhu H.H., Xu Q., Shi B., Mei G.X. (2014). Time-dependent pullout behavior of glass fiber reinforced polymer (GFRP) soil nail in sand. Can. Geotech. J..

[16] Hong C.Y., Yin J.H., Zhou W.H., Pei H.F. (2011). Analytical study on progressive pullout behavior of a soil nail. J. Geotech. Geoenviron. Eng..

[17] Schilder C., Kohlhoff H., Hofmann D., Basedau F., Habel W.R., Baebler M., Herten M. Static and dynamic pile testing of reinforced concrete piles with structure integrated fibre optic strain sensors. Proceedings of the Fifth European Workshop on Optical Fibre Sensors, International Society for Optics and Photonics.

[18] Lee W., Lee W.J., Lee S.B., Salgado R. (2004). Measurement of pile load transfer using the Fiber Bragg Grating sensor system. Can. Geotech. J..

[19] Kong X., Cai C.S., Hou S. (2013). Scour effect on a single pile and development of corresponding scour monitoring methods. Smart Mater. Struct..

[20] Wang H.L., Peng L., Zhao Z.G., Li Y.N., Li C., Peng Q.J., Wang D.D. (2015). On the application of fiber Bragg Grating strain piles for monitoring the slope of mountain substations. Proceedings of the 2nd International Conference of Structural Health Monitoring and Integrity Management (ICSHMIM 2014).

[21] Zhu H.H., Yin J.H., Jin W., Zhou W.H. Soil nail monitoring using Fiber Bragg Grating sensors during pullout tests. Proceedings of the Joint 60th Canadian Geotechnical and 8th IAH-CNC Conferences.

[22] Zhu H.H., Yin J.H., Yeung A.T., Jin W. (2010). Field pullout testing and performance evaluation of GFRP soil nails. J. Geotech. Geoenviron. Eng..

[23] Hong C.Y., Yin J.H., Jin W., Wang C., Zhou W.H., Zhu H.H. (2010). Comparative study on the elongation measurement of a soil nail using optical lower coherence interferometry method and FBG method. Adv. Struct. Eng..

[24] Pei H., Yin J., Zhu H., Hong C. (2012). Performance monitoring of a glass fiber-reinforced polymer bar soil nail during laboratory pullout test using FBG sensing technology. Int. J. Geomech..

[25] Li G.W., Pei H.F., Hong C.Y. (2013). Study on the stress relaxation behavior of large diameter B-GFRP bars using fbg sensing technology. Int. J. Distrib. Sens. Netw..

[26] Jin Q.P., Zheng Z.J., Dou B.Q., Lei X.W. (2014). FBG Sensor Application for GFRP Soil Nailing Pull-Out Test. Appl. Mech. Mater..

[27] (2013). ASTM D4435-13e1, Standard Test Method for Rock Bolt Anchor Pull Test.

[28] Ansari F., Libo Y. (1998). Mechanics of bond and interface shear transfer in optical fiber sensors. J. Eng. Mech..

[29] Won J.P., Park C.G., Kim H.H., Lee S.W., Jang C.I. (2008). Effect of fibers on the bonds between FRP reinforcing bars and high-strength concrete. Compos. Part B Eng..

[30] Sawicki A. (2000). Mechanics of Reinforced Soil.

